# Roles for Microglia in Cryptococcal Brain Dissemination in the Zebrafish Larva

**DOI:** 10.1128/spectrum.04315-22

**Published:** 2023-01-31

**Authors:** Jacquelyn A. Nielson, J. Muse Davis

**Affiliations:** a Stead Family Department of Pediatrics, Carver College of Medicine, University of Iowa, Iowa City, USA; Mycology Laboratory, Wadsworth Center

**Keywords:** *Cryptococcus*, blood-brain barrier, endothelial cells, host-pathogen interactions, macrophages, microglia, pathogenesis

## Abstract

Cryptococcal infection begins in the lungs, but yeast cells subsequently access the bloodstream, from which they can reach the central nervous system (CNS). The resulting meningoencephalitis is the most common presentation and is very difficult to treat. How this fungus interacts with the blood-brain barrier (BBB) and establishes growth in the brain parenchyma remains a central question in fungal pathogenesis. We and others have developed the zebrafish larva as a model host for cryptococcosis and demonstrated that hematogenous CNS infection is replicated in this model. Here, we have used this model to examine the details of BBB crossing and the events immediately before and after. We have observed multiple mechanisms of BBB crossing and found that microglia, the resident phagocytes of the brain, likely have multiple roles. First, microglia either actively transfer yeast cells across the BBB or take up a significant proportion of them immediately after crossing. Second, microglia are capable of clearing individual cryptococcal cells at a developmental stage before adaptive immune cells have emerged. Third, microglia serve to maintain endothelial integrity, preventing other, phagocyte-independent forms of crossing. These proposed microglial functions during infection in the zebrafish larva generate new hypotheses concerning the establishment and control of cryptococcal meningoencephalitis.

**IMPORTANCE** Cryptococcal meningitis is a fungal infection of the brain and a major cause of death in people with uncontrolled HIV. Infection begins in the lungs but can enter the bloodstream and disseminate to the brain. A structure called the blood-brain barrier must be crossed for the fungus to enter and cause meningitis. Learning how Cryptococcus crosses the blood-brain barrier will be crucial to understanding and possibly preventing brain infection. Using the zebrafish larva as a model host, we show that microglia, the resident phagocytes of the brain, potentially play multiple previously unappreciated roles in cryptococcal infection of the brain. These roles include reinforcing the integrity of the blood-brain barrier, clearing cryptococcal cells after they have crossed, and possibly participating directly in crossing via a previously unknown mechanism.

## INTRODUCTION

The most common presentation of cryptococcal infection is meningoencephalitis, which can take on multiple pathological forms depending on host immune status (1). By the time central nervous system (CNS) infection becomes symptomatic, it is already exceedingly difficult to treat. The initial crossing of what is broadly called the blood-brain barrier (BBB) by Cryptococcus is thus a clinically silent but extremely consequential event. Multiple mechanisms for cryptococcal BBB crossing have been proposed, each with various amounts of *in vitro* and *in vivo* evidence (2–7). Two general schemes have emerged. In the first scheme, yeast cells are carried across the BBB within macrophages (the so called “Trojan Horse” mechanism) (8–10). In the second scheme, yeast are either actively taken up by the luminal endothelium and expressed out the other side (transcytosis) (2, 4, 11) or they directly cross the BBB between joined endothelial cells (paracellular movement) (12).

A major barrier to studying BBB crossing is the rarity of the event. For example, *in vitro* studies using an endothelial monolayer in a transwell found that only 1 to 2% of yeast cells are able to cross (7). *In vivo* visualization of crossing in the mouse has been accomplished but has been difficult to expand on in part because, again, crossing events are rare (4). An extensive study of brain pathology in mice (13) has given us good insight into the microanatomy of infection but could not demonstrate the process in living tissue. Further complicating the problem, both *in vitro* and *in vivo* investigations apply a very large number of yeast either to a monolayer or directly into the bloodstream. It is not clear how relevant such a large yeast burden is to human pathophysiology. Although Cryptococcus cells certainly cross the BBB in these models, the low efficiency of the phenomenon has made it difficult to determine the relative importance of different mechanisms.

*In vitro* models of cryptococcal BBB crossing have thus far failed to recapitulate the complexity of the BBB. In its general form, the barrier is composed of multiple cell types besides endothelial cells, including astrocytes, pericytes, neurons, and microglia, the resident phagocytes of the brain parenchyma (14). All these cells play important roles in supporting BBB function and integrity. It is unlikely that Cryptococcus cells gain access to the brain parenchyma without encountering these cell types along the way. Indeed, posthumous observation of brain tissue both from mice and from human AIDS patients with cryptococcal meningitis revealed activated astrocytes and microglia surrounding cerebral lesions (15). Although recent “organ-on-a-chip” models have successfully incorporated multiple neurovascular cell types in an *in vitro* model of cryptococcal infection (16), this technology requires further validation and remains technically demanding. To understand the mechanisms behind cryptococcal meningoencephalitis, we need a way to generate testable hypotheses about the roles of different cells, roles that can be examined with relevant cell types alone and in combination using the different available models.

Using the zebrafish model of cryptococcal infection, we and others have established many parallels in pathogenesis between this model and mammalian models (17–19). A key advantage of the zebrafish is that its small size and transparent tissues allow the tracking of pathogenesis using a small inoculum (e.g., 30 to 70 yeast cells) over multiple days. Doing so, we have previously shown that yeast introduced via the caudal vein are completely taken up by phagocytes during the first hours of infection (17). After hours to days of intracellular survival, very few yeast cells escape into the bloodstream (a phenomenon we call “secondary fungemia”), so few at first that even continuous monitoring reveals only intermittent findings of free yeast. In keeping with this finding, fungemia is rarely detected in samples from presymptomatic patients (20).

Zebrafish larvae develop a BBB similar to that of mammals, incorporating all of the aforementioned cell types of the neurovascular unit. By 3 days postfertilization (dpf), the zebrafish brain microvasculature is capable of restricting large molecules (such as 2,000-kDa dextran) (21) and can exclude smaller molecules (such as the 961-Da Evans Blue) (22) by 5 dpf. This exclusion is supported by the presence of the tight junction proteins zonula occludens-1 (ZO-1) and claudin-5, which can be found in the larval BBB as early as 3 dpf (21). The earliest macrophages of the zebrafish derive from the ventrolateral mesoderm, initially migrating to the lateral sides of the yolk (Fig. S1A in the supplemental material) (23). A subset of these cells migrates into the head mesenchyme and differentiates into the early population of microglia by 3 dpf (Fig. S1B) (24). Genetic profiling confirms that these cells share a conserved genetic signature with mouse and human microglia (25). Thus, the easily imaged neurovascular environment of larval zebrafish at 2 to 7 dpf has many similarities to the relevant tissues in humans (Fig. S1C).

In the zebrafish, hematogenous spread into the CNS by Cryptococcus has been documented by us and others (17, 19), but the model has not yet been fully used for studying this process. In our prior studies, we found that during secondary fungemia, yeast cells that exit phagocytes and reach the blood had multiple fates, including entry into the CNS and subsequent growth there (17). Here, we have pursued the hypothesis that this low level “secondary fungemia” is the source of CNS infection in humans and have focused on the fates of the yeast cells that reach the brain. By compiling a large number of live observations over multiple days, we have quantified multiple aspects of CNS infection *in vivo*. As in previous studies, we found evidence for multiple independent mechanisms for BBB crossing, although no single mechanism is essential. Because the long-sought-after key to preventing BBB crossing altogether remains elusive, we have broadened our focus to examine CNS infection before and after that event. The zebrafish model is uniquely well suited for this purpose.

Our observations suggest the hypothesis that the yeast-endothelium interaction precedes and influences all crossing events and may on its own result in crossing of the BBB. Alternatively, phagocytes can interrupt this interaction and carry yeast across themselves, and we present evidence for a novel phagocyte-mediated mechanism in which microglia participate in the process. By following the outcome of BBB crossing, we find that the mechanism of crossing may influence subsequent disease progression. Our *in vivo* observations provide a new perspective on the early steps of cryptococcal meningoencephalitis and underscore the relevance of the multicellular nature of the BBB in models of Cryptococcus pathogenesis. Microglia in particular can play multiple roles, including mediating yeast crossing directly, supporting the integrity of the vascular endothelium, and clearing yeast cells after crossing, even in an organism that does not yet have a functional adaptive immune system. The evolutionary distance between zebrafish and mammals means that we must explore and confirm these findings. However, the unprecedented degree of visual access to brain dissemination provided by this model provides new hypotheses to test in more familiar models.

## RESULTS

### The majority of yeast reaching the brain are extracellular.

**FIG 1:**
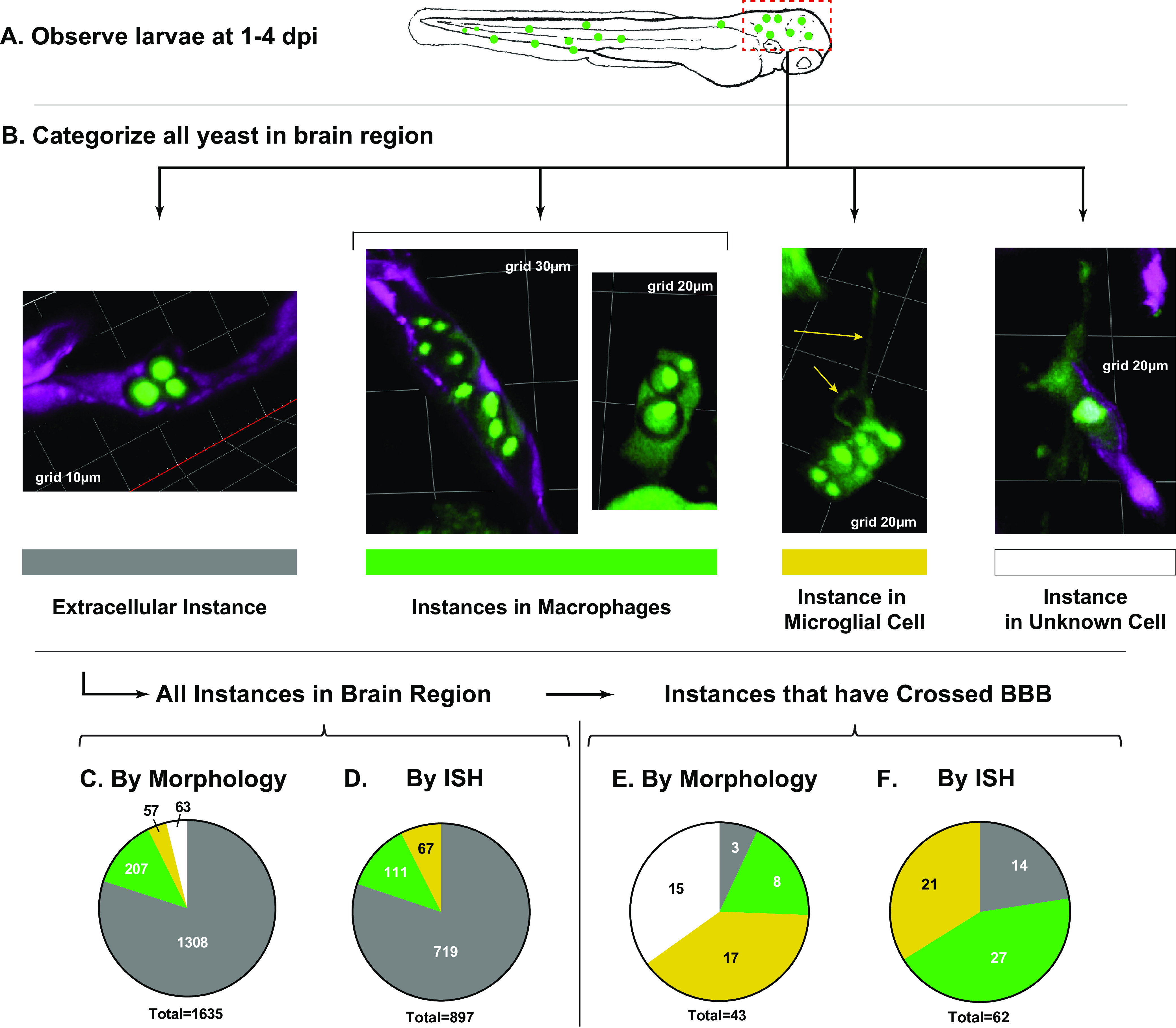
Quantification of cryptococcal yeast cells in the brain from 1 to 4 dpi. (A) Schematic workflow. Instances of cryptococcal yeast in the brain were quantified in observation and enumerated in the brains of a total of 77 live larvae compiled in 3 replicates. (B) Instances were categorized based on morphology. Note that cells with macrophage morphology are seen both inside and outside the vasculature. (C) Morphological quantification of all brain instances in live larvae. Colors correspond with the bars in B; gray, extracellular; green, macrophages; yellow, microglia; white, uncertain morphology. (D) Quantification of all brain instances in a separate set of fixed larvae using ISH for positive identification of microglia. (E) Quantification of the subset of instances that have crossed the BBB in the live larva set. (F) Quantification of the subset of instances that had crossed in the fixed larva set.

To quantify cryptococcal BBB crossing in zebrafish larvae, we infected double transgenic Tg(*mpeg1:EGFP*) × Tg6(*kdrl:mCherry*) zebrafish intravenously with ~30 to 70 C. neoformans yeast cells. The double transgenic larvae express enhanced green fluorescent protein (EGFP) in the cytoplasm of macrophages and microglia and mCherry in the cytoplasm of endothelial cells. We will refer to these larvae as *mpeg/kdrl* fish. The cryptococcal strain used (JMD163) is a KN99 strain expressing nuclear-localized EGFP, giving the yeast cells a pinpoint appearance that is easily distinguishable from host-expressed EGFP (as seen in the images of Fig. 1). Compiling a large number of observations, we generated a control database from 3 separate replicates of a total of 77 infected larvae. Each larva was observed daily from 1 to 4 days postinfection (dpi) (Fig. 1A), and yeast cells in the brain (both intravascular and extravascular) were characterized (Fig. 1B). To relate our counts to potential instances of crossing, we counted any cluster of yeast as a single “instance,” as a tight cluster is more likely to represent a single potential crossing event instead of multiple ones (as exemplified in Fig. 1B). We counted 1,635 instances of Cryptococcus yeast cells in the brain among three replicates for an average of 21 instances per fish. We categorized these instances of yeast into four distinct categories, shown in Fig. 1B: (i) extracellular, (ii) in a cell with macrophage morphology, (iii) in a cell with microglial morphology, and (iv) in a cell that could not be confidently called macrophage or microglia based on morphology alone. In all three replicates combined, 80.0% of instances were extracellular yeast (Fig. 1C, gray), while the remainder were split between macrophages (green), microglia (yellow), and cells of indeterminate morphology (white). Charts of the individual replicates are shown in Fig. S2 in the supplemental material.

To distinguish macrophages and microglia definitively, we used *in situ* hybridization (ISH) for apolipoprotein E (*apoE*) RNA. *apoE* expression has previously been used as a marker for early microglia in the zebrafish larva (24). Using this technique in Tg(*mpeg1:EGFP*) larvae at 1 to 4 dpi, we determined that the majority of EGFP^+^ cells in the brain also expressed apoE (Fig. S3A), and the morphology of these cells (infected and uninfected) matched the highly ramified EGFP^+^ cells seen in living larvae (Fig. S3B). To apply this method to daily observations of infection, we performed a separate experiment in which we sampled a subset of infected larvae each day for *in situ* hybridization. Of 897 instances in this analysis, 719 were extracellular and 178 were intracellular, comparable to our findings in live fish (Fig. 1D).

### Yeast that have crossed the BBB are predominately intracellular.

To learn more about the specific conditions required for yeast to cross the BBB, we returned to the original large data set of 1,635 instances and focused on the 43 instances observed in the brain parenchyma. In contrast to the total instances, we found that a large majority of these were inside macrophages or microglia (Fig. 1E). Analysis of infected larvae fixed and probed for *apoE* expression showed that crossing instances were divided roughly equally between macrophages and microglia (Fig. 1F). Based on our findings thus far, we surmised the following for this model: (i) because it is extremely rare to observe infected macrophages circulating in the blood and the vast majority of intravascular yeast were extracellular, the first step of brain dissemination is the arrival of extracellular yeast in the vasculature as a feature of “secondary fungemia” (17); (ii) among these intravascular yeast, only a very small proportion (~2.6%) cross the BBB in the first 4 days of infection; and (iii) most yeast that have crossed the BBB are inside macrophages or microglia and crossed either inside the cells or were taken up shortly after crossing by other means.

To determine the role of vascular integrity in BBB crossing, we injected 10-kDa dextran labeled with a far-red fluorescent dye into the vasculature at 4 dpi (6 dpf) and imaged instances of Cryptococcus in the brain. Dextran of this mass is known to be excluded from the brain in uninfected larvae at this stage (21). We found that in brains with a high cryptococcal burden, some leakage was visible (Fig. S4A), but yeast could readily be found in the parenchyma without evidence of leakage (Fig. S4B). We interpreted this to mean that large quantities of Cryptococcus could impair the chemical BBB, but such impairment was not necessary for CNS dissemination to occur. This is in keeping with findings *in vitro*, showing that yeast crossing an endothelial layer on a transwell do not disrupt the electrical potential across the endothelium (7).

### Phagocytes facilitate BBB crossing by a cryptococcal mutant that does not cross alone *in vitro*.

**FIG 2:**
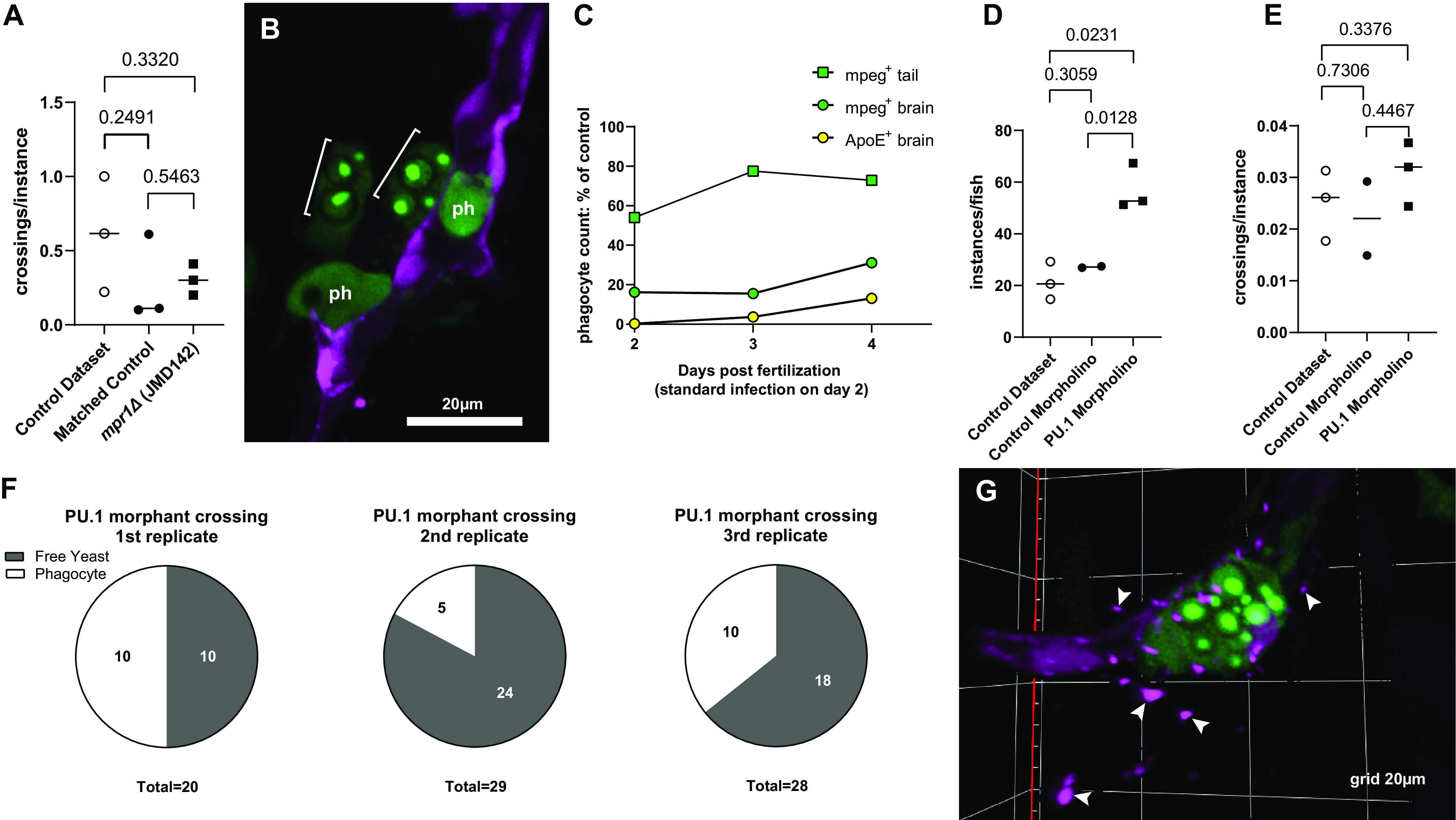
(A to F) Analysis of brain instances in *mpr1*Δ infection (A and B) and in PU.1 morphant larvae (C to F). (A) Crossing efficiency (number of crossings per instance in the whole experiment) in the original control data set of 77 larvae, matched control (JMD163) infection (closed circle), and *mpr1*Δ infection (closed square). The latter 2 sets were obtained simultaneously and consisted of 3 replicates of 10 larvae each. Pairwise analyses throughout the figure represent Welch’s *t* test of log-transformed ratios. (B) Intracellular *mpr1*Δ yeast cells (brackets) in the parenchyma after crossing the BBB; Ph, uninfected phagocytes. (C) Percent reduction in phagocytes in PU.1 morphants compared to larvae that received control morpholino. All mpeg^+^ cells decreased in the brain, but apoE^+^ cells decreased more. (D and E) Instances per fish (D) and crossing efficiency (E) comparison between the original control data set of 77 larvae, control morpholino (2 replicates of 10 larvae each), and PU.1 morpholino (3 replicates of 16, 15, and 13 larvae). (F) Status of crossing instances in PU.1 morphant infections; *n* = 3 replicates as in C and D; gray, extracellular; white, intracellular. (G) Example of endothelial breakdown in the brain during infection of PU.1 morphant. Infected Phagocyte inside a deteriorating vessel. White arrowheads indicate fragments of endothelial cytoplasm.

To assess the possibility of phagocyte-mediated crossing, we used JMD163 to produce three independently generated cryptococcal mutant strains lacking metalloproteinase 1 (*mpr1*Δ strains: JMD142, JMD154, and JMD155). An H99 strain lacking this gene is defective in phagocyte-free crossing of an endothelial layer *in vitro* (26). We predicted that if brain phagocytes carry yeast out of the vasculature in zebrafish, the *mpr1*Δ mutant would still cross the BBB. Our prediction was correct. We observed an average of 0.30, 0.21, and 0.55 crossings per fish for infections with the *mpr1*Δ mutants JMD142, JMD153, and JMD155, respectively. This was comparable to that observed for infections with the parental JMD163 strain (0.27) and in the control data set (0.57). Crossing efficiency (crossing instances as a percentage of total brain instances) was not significantly different for the *mpr1*Δ mutant JMD142 relative to either set of control data (Fig. 2A). The observation of BBB crossing by the *mpr1*Δ mutant (Fig. 2B) confirms that mechanisms other than transcytosis (for which the gene is required) contribute to brain dissemination in the zebrafish model.

### Both phagocyte types influence the mode of BBB crossing.

To investigate the relative importance of phagocyte-mediated BBB crossing, we used a well-established method for reducing macrophage (and microglia) populations in the zebrafish. PU.1 is a myeloid transcription factor required for early larval phagocyte production. Transient knockdown of this gene results in delayed macrophage development. Because microglia are derived from the very first macrophage progenitors early in development (24), PU.1 knockdown greatly reduces the establishment of microglia in larval brain tissue. Knockdown is accomplished by microinjecting PU.1-targeted morpholinos into the zebrafish embryo at the one-cell stage of development. Morpholinos are durable RNA-like molecules targeted to pair with specific RNA to prevent splicing and/or translation of specific genes (27). A standard control morpholino known to cause minimal changes in gene expression was used for comparison. For knockdown of PU.1, we used a previously described pair of morpholinos, one targeting the transcription initiation site and one targeting the exon 4 to 5 boundary (27). We confirmed the efficacy of this morpholino pair by injecting *mpeg*/*kdrl* embryos with morpholinos at the one-cell stage and counting EGFP^+^ cells daily from 2 to 6 days postfertilization in the resulting larvae (morphants). Phagocyte depletion was greater in the brain than in the tail (Fig. S5A and B). To confirm the effect of these morpholinos on the microglial population specifically, we again used *in situ* hybridization for apolipoprotein E in uninfected morphants. By counting mpeg^+^/ApoE^+^ double-positive cells, we found that while there were some *apoE*-expressing microglia in the brain, they were depleted to a greater extent (99.7 to 86.9%) than macrophages (83.8 to 68.9%) (Fig. 2C). Therefore, in these PU.1 morphants, microglia are very much depleted, and macrophages are less so.

Infection of PU.1 morphants with JMD163 progressed faster than in controls (data not shown), so a smaller inoculum of 15 to 30 yeast cells was used in these larvae to prevent overwhelming fungemia during the observation period. Relative to control morpholino fish and our original control data set, total brain instances per fish increased in the PU.1 morphant (Fig. 2D). We attributed this to a whole-larva increase in extracellular yeast, leading to more instances of extracellular yeast lodging in the brain vasculature. Crossing efficiency was unchanged (Fig. 2E), suggesting that BBB crossing does not rely entirely on phagocyte-mediated mechanisms. Analyzing the instances of yeast that had crossed the BBB, we found that the majority were extracellular (Fig. 2F), suggesting that while phagocytes do contribute to BBB crossing, phagocyte-independent crossing is increased in their absence. This increase could be due to poor cryptococcal control by a reduced number of macrophages or to breakdown of stressed endothelium. Microglia are known to have a role in maintaining endothelial integrity (28), and evidence of endothelial breakdown during infection of PU.1 morphants was apparent in some larvae (~3 to 4 fish) in our observations (Fig. 2G).

**FIG 3:**
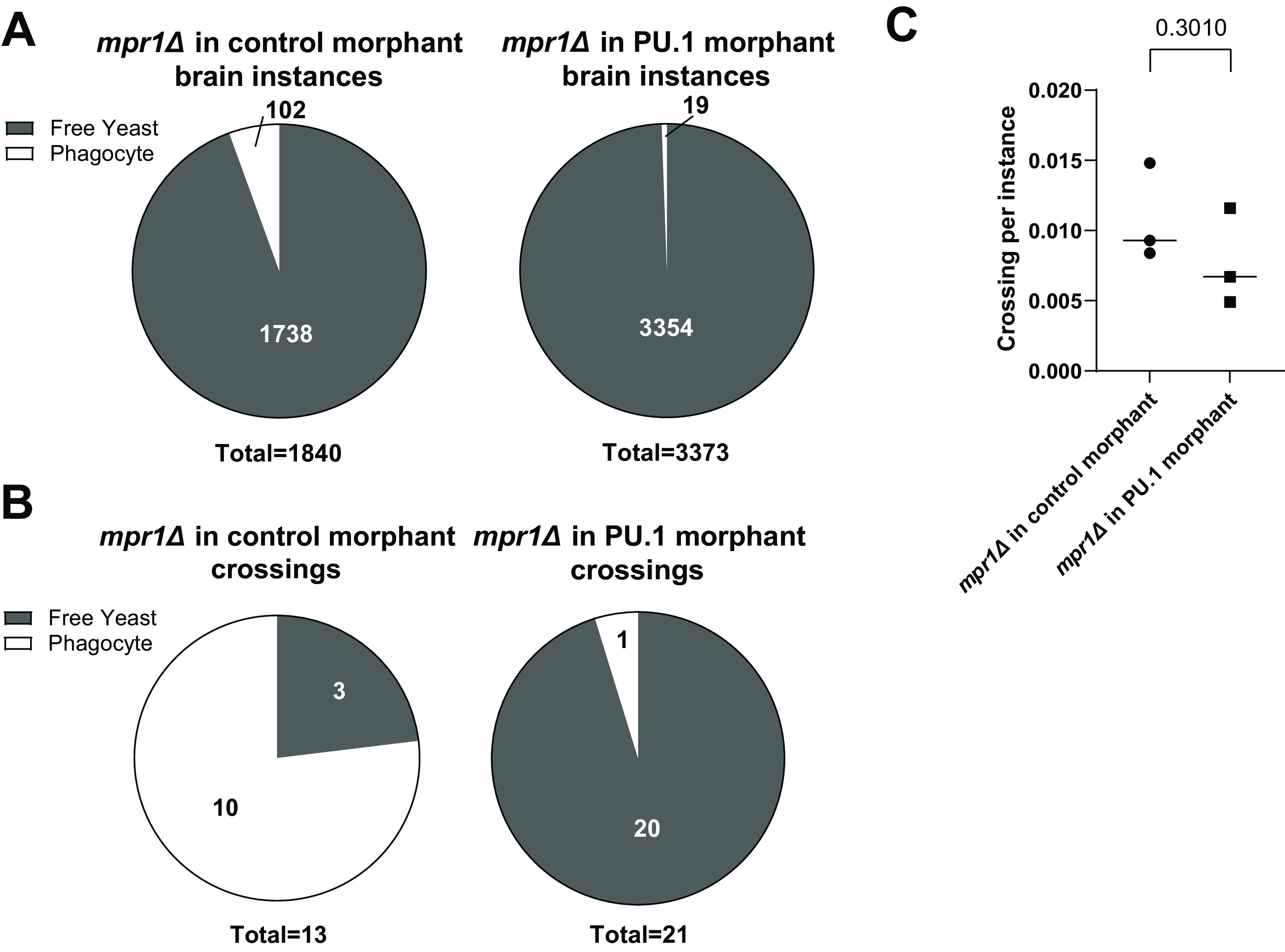
Quantification of *mpr1*Δ infection of control versus PU.1 morphants. (A) Total brain instances extracellular (gray) and intracellular (white); *mpr1*Δ + control morphant, *n* = 3 replicates of 10 larvae each; *mpr1*Δ + PU.1 morphant, *n* = 3 replicates of 9, 10, and 10 larvae. (B) Status of crossing instances from same replicates as A. (C) Crossing efficiency in same replicates as A and B. Pairwise analyses throughout the figure represent Welch’s *t* test of log-transformed ratios.

To determine if the relative increase in phagocyte-independent crossings was due to a process other than transcytosis, we infected PU.1 morphants with the *mpr1*Δ mutants and compared them to control morphants also infected with the *mpr1*Δ mutant. Brain instances in phagocytes decreased even more (Fig. 3A), and *mpr1*Δ yeast that had crossed in PU.1 morphants were overwhelmingly extracellular (Fig. 3B). This is consistent with contributions by at least one Mpr1-independent extracellular crossing mechanism (such as endothelial breakdown) in the zebrafish, mediating crossing when transcytosis is not possible. *mpr1*Δ crossing efficiency in PU.1 morphants was not significantly different from wild type (Fig. 3C), suggesting that the alternative crossing mechanism(s) have similar combined capacity to that of transcytosis.

### Both macrophages and microglia participate in dissemination to the brain.

**FIG 4:**
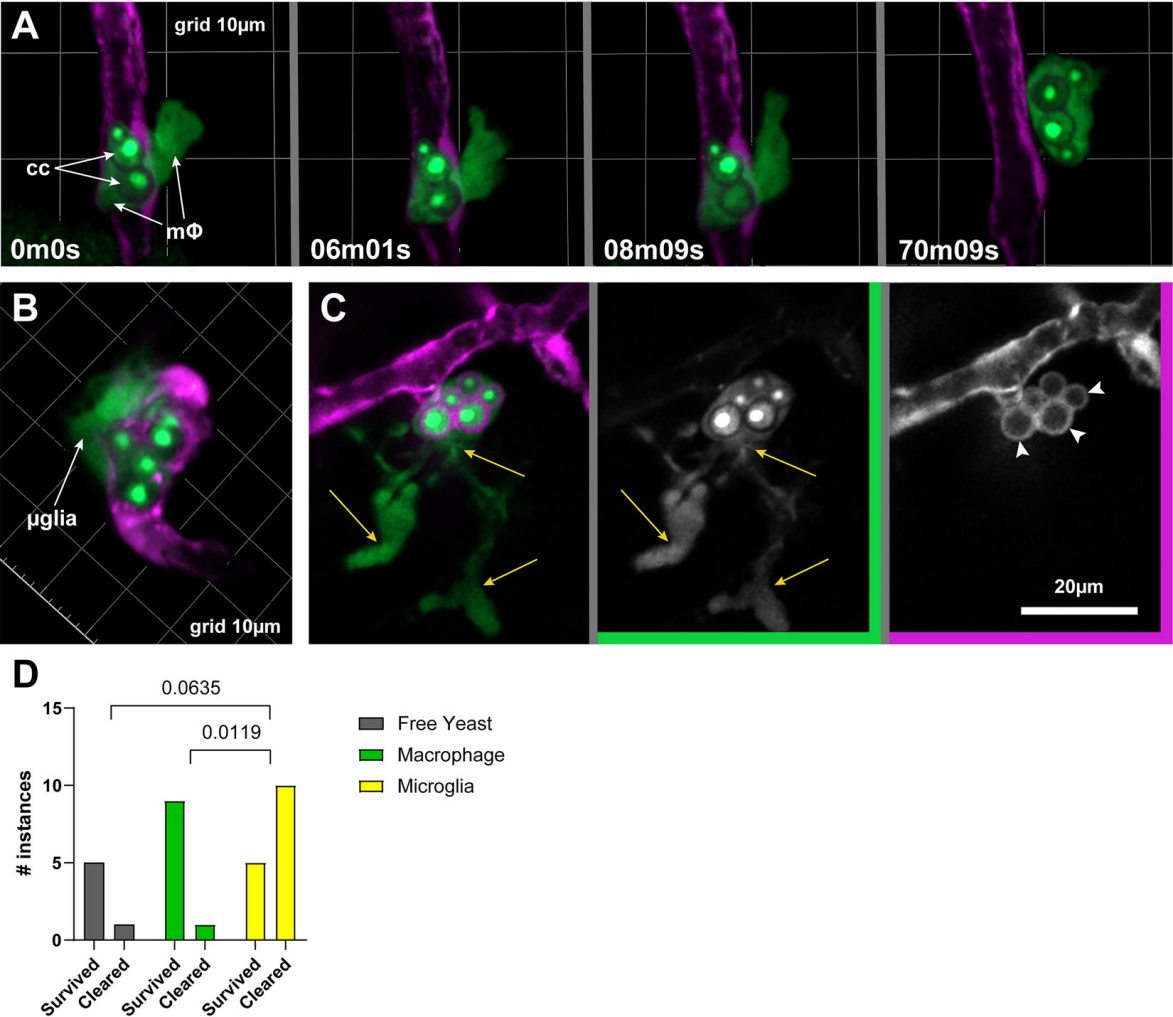
Macrophage and microglia in BBB crossing and subsequent fate of Cryptococcus. (A) Intravascular macrophage (mΦ) containing cryptococcal yeast cells (cc) extends a pseudopod outside the vasculature and pulls its cargo along with it. Phagocytes (mpeg^+^) and yeast cells express EGFP. Note that nuclear-localized fluorescence signal emphasizes yeast cells versus phagocytes of the same color. Endothelial cells express cytoplasmic mCherry (magenta). (B) A presumed microglial cell (μglia) tightly associated with the abluminal side of a vessel containing Cryptococcus. (C) A cell with microglial morphology in the parenchyma (yellow arrows indicate ramifications) containing yeast cells along with endothelial cytoplasm (white arrowheads). (D) Fate of crossing instances compiled from the original control data set plus additional replicates performed specifically for this analysis. *P* values indicate results of a Fisher’s exact test applied to contingency data.

Based on our findings thus far, both macrophages and microglia may play an active role in BBB crossing in this model. To understand these roles at a mechanistic level, we carefully observed the cellular events in the brains of infected larvae daily from 1 to 4 dpi. Evidence for two mechanisms of phagocyte-mediated BBB crossing was observed. First, an infected macrophage in the vasculature extended a pseudopod out into the parenchyma and carried the Cryptococcus across (Fig. 4A). Second, microglial cells (based both on morphology and *apoE* expression) arrive at points in the vasculature where cryptococcal cells are lodged (Fig. 4B). They are later seen, still in the parenchyma, containing yeast (Fig. 4C). This observation could also be due to microglia engulfing yeast cells after they have crossed on their own, but these data also raise the novel possibility that microglia act directly to bring Cryptococcus across the BBB. In many such cases, the mCherry signal of endothelial cytoplasm forms a sphere that encircles phagocytosed C. neoformans (Fig. 4C, arrowheads). This is consistent with either simultaneous phagocytosis of yeast and the endothelium around it or with separate uptake of yeast cells and endothelial vesicles, both of which are then targeted to the same compartment. The suggestion that microglia may actively participate in BBB crossing is tantalizing, but regardless of the crossing mechanism, the fact that so many yeast cells end up inside this cell type after crossing calls for a better understanding of their response.

### Phagocyte types encountered during and after BBB crossing impact the fate of Cryptococcus in the brain.

Regardless of how crossing ultimately takes place, we wanted to understand the implications of microglial involvement with yeast in the parenchyma. Previous studies of mycobacterial infection in the zebrafish demonstrate that the “native” phagocytes of the brain are less permissive to microbial growth and survival than phagocytes recruited from the bloodstream (29). To determine if this was true in the case of cryptococcal infection, we compiled crossing instances from several JMD163 infection data sets and combined them with additional replicate infections specifically focused on this question and analyzed the survival of yeast over a 24-h period after they had crossed the BBB. Survival was assessed by the presence or absence of green fluorescent signal and was correlated with differential interference contrast (DIC) imaging. The EGFP expressed by our marked strains bears a nuclear localization signal, and in healthy cells, the protein is highly concentrated in the nucleus. We have shown that dissipation of EGFP signal is an early sign of cryptococcal clearance (17). Because of how rare crossing events are, and the fact that we could only analyze instances that had happened early enough for follow-up, our data set was quite small. Of 6 free yeast cells followed, only 1 cell was cleared. Results were similar for yeast seen in macrophages; of 10 instances, again only 1 was cleared. In contrast, 10 of the 15 instances in microglia were cleared, suggesting that regardless of crossing mechanism, microglia serve a protective role in this model. A contingency analysis of these results is shown in Fig. 4D. Prior *in vitro* studies have suggested that isolated microglia do not clear cryptococcal cells very well (30). It is unclear whether the different results arise from supportive signals from other cell types *in vivo*, from changes in the capacity of microglial cells when isolated, or from factors particular to our model. In the zebrafish model, at least, in addition to supporting endothelial integrity (a known role in mammalian models [31, 32]) and possibly playing a role in BBB crossing, microglia can be host protective in cryptococcal infection by directly killing yeast cells in an organism that does not yet have a functional adaptive immune system.

## DISCUSSION

CNS infection is the most lethal manifestation of cryptococcal infection and a leading cause of mortality in people living with HIV (33). We know that yeast cells arrive via the bloodstream and can replicate in the parenchyma. In patients with poorly controlled HIV, this replication is mostly extracellular, while it is mostly inside phagocytes in non-HIV cases (1). A great amount of work has been done to understand the first step of infection, the molecular mechanism of BBB crossing. In zebrafish, as stated in previous literature, evidence suggests that no single dominant mechanism of crossing exists (2–6, 9). Rather, multiple complementary mechanisms are in use. We hypothesize that the direct interaction between Cryptococcus and the endothelium is the key initiating event for multiple mechanisms. In addition, we have used the zebrafish model to make observations not only about crossing but also about subsequent CNS pathogenesis. We find evidence for multiple potential roles for microglia.

Starting with BBB crossing, proposed phagocyte-independent crossing mechanisms include transcytosis (requiring Mpr1), paracellular movement, and a recently described hemorrhagic mechanism (34). In our zebrafish studies, hemorrhagic crossing becomes conspicuous in the absence of endothelial support by microglia, but its role relative to transcytosis could not be assessed. Our findings raise the novel possibility that there are two phagocyte-dependent mechanisms, involving either circulating macrophages or resident microglia. We have been unable to test the role of macrophages in BBB crossing directly, as their complete absence alters the course of pathogenesis in favor of overwhelming infection (35). Even so, macrophage-mediated crossing appears to be quite common in this model. Regardless of whether microglia actively bring the yeast across the BBB or simply capture them after they arrive, our finding of a potentially more host-protective role for microglia than for macrophages is intriguing.

Based on our findings, we propose that the first step of BBB crossing in this model is the arrival of free yeast in the CNS vasculature and that this is common to all crossing mechanisms. If these yeast dwell long enough, they begin to interact with the endothelium in a process that can lead to transcytosis, as seen *in vitro* (6, 7, 9). However, the process is frequently interrupted by either macrophages or potentially microglia, which, in turn, facilitate BBB crossing by other mechanisms. Early events in transcytosis could even be triggering these other mechanisms by recruiting and activating phagocytic cells.

Another implication of these findings is that resident microglia have at least three potential roles in cryptococcal pathogenesis: (i) supporting the integrity of the BBB itself, as has been demonstrated in other contexts (28, 32); (ii) restricting yeast growth within the CNS; and (iii) actively bringing yeast cells across the BBB. In humans, microglia are known to be infected by and to act as a major reservoir for HIV, becoming senescent as HIV infection lingers (36–38). Thus, it is not at all incongruent to propose such important roles for this cell type specifically for controlling cryptococcal dissemination.

Modeling the interface between the central nervous system and the circulation is extremely difficult. Even the phrase “blood-brain barrier” is a vast oversimplification of the complex of cell and tissue types relevant to microbial dissemination into the brain. The term “meningoencephalitis” is used in the context of cryptococcosis due to involvement of both meninges and the parenchyma of the brain. The vasculature of the meninges in humans includes lymphatic vessels and the associated arachnoid space. Access to the parenchyma at the microvascular level occurs in different anatomical contexts, with a variety of immune cell types present (39, 40), and in the context of blood flow. The common factor throughout is the endothelium, although endothelial cells also vary in their characteristics depending on location (40). Therefore, any reductive approach to CNS dissemination by Cryptococcus must be interpreted with caution. Another universal challenge to the study of cryptococcal brain dissemination is how clearly rare crossing events are; this is consistent in our model, in mice, and *in vitro* (4, 7).

The larval zebrafish model is relatively new to the field of cryptococcosis but has the unique advantage of providing visual access to dissemination in the context of many of the relevant cells and tissues. Still, we must be clear about the caveats. A main advantage of the larval zebrafish is that it lives off its yolk for the first 7 days of life at least (41). After that point, providing consistent nutrition with good water quality is quite difficult even without daily handling and microscopic observations. Thus, a window of time from day 2 to day 7 is most ideal for observations. Two days postfertilization is still extremely early in the development of the brain and its vasculature, and the landscape and physiology of the blood-brain barrier changes daily. Although the brain vessels can exclude larger molecules by 3 dpf, the exclusion of smaller molecules observed in mature BBBs is not uniformly achieved until 10 dpf (22). Additionally, brain vessel angiogenesis and pruning are common during the time of infection, although apoptosis of endothelial cells and phagocyte interference is not associated with vessel pruning (42). Crucially, careful study in the mouse model raises the strong possibility that CNS dissemination in mammals takes place at least partially in the perivascular space of postcapillary venules (13) and not through the neurovascular unit (endothelium, astrocytes, and pericytes) that we associate with the chemical “blood-brain barrier.” This aspect of the microarchitecture is not replicated in the zebrafish larva and has not been included in *in vitro* models of dissemination either. We do propose that even without perivascular spaces, the larval zebrafish can generate testable hypotheses regarding other cell types present around the vasculature of the brain. Finally, the behavior of larval microglia differs somewhat from the later population of mature microglia, which appears around 20 dpf. Larval zebrafish microglia are more motile and phagocytic than mature microglia, adopting a more amoeboid morphology with only transient ramified processes (43). However, the early microglia become less motile and more ramified with time, adopting a gene expression profile comparable to adult mouse and human microglia by 5 dpf (3 dpi) (25).

In other models, the key challenge to understanding CNS infection by Cryptococcus is the relative rarity of the key events. This limitation is no less true for the zebrafish larva. While we have amassed and analyzed a large number of observations, our findings do not generate the statistical power that we would like. However, the model still provides an exceptionally close look at potentially relevant phenomena in the context of an unprecedented variety of relevant cell types. As such, the hypothesis-generating capacity of the model may be its best contribution to this question. A number of testable hypotheses can be generated from our findings and examined using other approaches. Key questions raised by our findings include, if the endothelium-yeast interaction recruits and activates macrophages and microglia, what signals evoke this response? What contributions to this recruitment might astrocytes and pericytes make? What molecular signals drive the apparently enhanced clearance capacity of resident microglia? Can these be manipulated therapeutically? How do these results fit into the schema of inflammatory versus anti-inflammatory microglia phenotypes found in the context of adaptive immunity? A combination of approaches will be necessary for answering these questions.

## MATERIALS AND METHODS

### Zebrafish care and maintenance.

Adult zebrafish were kept under a light/dark cycle of 14 h and 10 h, respectively. Larval zebrafish were incubated at 28.5°C in E3 buffer (41), switching larvae to E3-methylen blue (MB) containing 0.2 nM 1-phenyl-2-thiourea (PTU; Sigma-Aldrich) at 18 to 24 h postfertilization (hpf) to inhibit pigment formation. All larvae were manually dechorionated between 24 and 30 hpf. Before microinjection or imaging, larvae were anesthetized in E3-MB containing 0.2 mg/mL tricaine (ethyl 3-aminobenzoate; Sigma-Aldrich). For prolonged time-lapse imaging, larvae were mounted in 1% low-melting-point agarose, 0.2 mg/mL tricaine, and 0.2 nM PTU (final concentrations) on a coverglass-bottom dish. All adult and larval zebrafish procedures were in full compliance with NIH guidelines and were approved by the University of Iowa Institutional Animal Care and Use Committee (protocol number 0102075-002).

### Transgenic zebrafish lines.

Tg(*mpeg1:EGFP*) is designated gl22Tg at the Zebrafish International Resource Center (ZFIN) and was kindly provided by Anna Huttenlocher. Tg6(*kdrl:mCherry*) is designated ubs42Tg and was originally provided by Heinz-Georg Belting and Markus Affolter. Transgenic lines are maintained by outcrossing to wild-type fish (strain AB) every other generation. ABs are obtained from ZFIN regularly to maintain hybrid vigor.

### Cryptococcal strains and growth conditions.

KN99α was kindly provided by Kirsten Nielsen. Cultures were handled using standard techniques and media, as described previously (44, 45).

### Construction of a nuclear-localized GFP expression strain.

pCH1227 (17) was provided by Christina Hull. A histone 3 promoter-driven eGFP with an 80-amino-acid nuclear localization sequence (NLS) and a nourseothricin (NAT) resistance cassette was amplified from pCH1227 using primers JMD01 (5′-GAG CTC GGC AGA TAC GAT ATG TTG G-3′) and JMD32 (5′-TAT CAT CAC GCC ACA CCC GG-3′). pH3mchSH2 (Addgene number 101053) (46) was used as a source for the SH2 flanking regions. The 5′-flanking region was amplified from pH3mchSH2 using primers JMD29 (5′-GAG CTC GGC AGA TAC GAT ATG TTG G-3′) and JMD28 (5′-tcg tat ctg ccg agc tCA AAA TCG TTG AAC CCG CAC-3′; lowercase letters indicate the overlap region for double joint PCR). The 3′-flanking region was amplified from pH3mchSH2 using primers JMD30 (5′-AGC GGA TAA CAA TTT CAC ACA GG-3′) and JMD33 (5′-ggt gtg gcg tga taA TCC GTC ACT CCT TTT TTC AGT C-3′). Products of these three reactions were combined in a double joint PCR, as described previously (47). The resulting construct was dubbed “Lodge Green” in honor of J.K. Lodge, the originator of pH3mchSH2. The Lodge Green construct was electroporated into KN99α as detailed previously (47). Colonies expressing green fluorescence were collected, and genomic DNA isolations were performed (48). The samples were then screened by PCR for proper insertion of the construct into the SH2 locus. Primer pair JMD34 (5′-CCT TCA ACA TTC TGA CGC TTT GTT TC-3′) and JMD 27 (5′-CGT TGT TTC AGG CCT GCG GAT G-3′) produce a 1,871-bp band from an intact SH2 locus or a 6,527-bp band from an SH2 site with the Lodge Green construct in place. This screen produced 2 positive candidates, which were compared with the wild type for growth in culture. The brighter of the two (JMD163) was used in the experiments here.

### Generation of a congenic *mpr1*-knockout strain.

Targeted gene knockout was performed using the transient CRISPR-Cas9 coupled with electroporation (CRISPR-TRACE) approach (47). Briefly, the genetic locus (CNAG04735) was subjected to a BLAST search against the published KN99 genome (tech) to identify the target locus in JMD163. This was analyzed using the Eukaryotic Pathogen CRISPR guide RNA/DNA design tool (http://grna.ctegd.uga.edu/) to locate potential unique target sites. One highly ranked 20-mer site (GCTTGGGCTTGAGAGTGTGG) was chosen due to its location in the gene. Constructs encoding single guide RNA (sgRNA) directed at this site and an insert containing a nourseothricin (NAT) resistance cassette flanked by ~900 bp of flanking sequence on each side were generated. These reagents were used for CRISPR-TRACE, as described previously (32). Resultant NAT-resistant colonies were passaged three times on plain yeast extract-peptone-dextrose (YPD) medium and rechallenged on YPD-NAT to ensure stability of the resistance marker. DNA was extracted from the resulting candidate colonies and screened by PCR according to the scheme in Fig. S6 in the supplemental material. Three strains (JMD142, JMD154, and JMD155) passed screening and were used to infect zebrafish larvae.

### Microscopy.

Confocal imaging was performed on a Zeiss Axio Observer.Z1/7 body equipped with a Zeiss Airyscan detector. All channels were collected using Airyscan Multiplex settings with default processing. Images in Fig. 4A were captured using a Zeiss LD C-apochromat ×40/1.1 W Korr UV-visible-infrared (UV-VIS-IR) objective. All other images were captured using a Zeiss Plan-apochromat ×20/0.8 objective. Live imaging was performed with larvae anesthetized under tricaine as previously described (49) and simply resting on the bottom of a glass-bottom dish or immobilizing in 1% low-melt agarose. For collection of large numbers of events, a combination of widefield and confocal imaging was used to create scout and detail images for later analysis. Widefield imaging was performed using the same Zeiss optical setup, with image capture using a Hamamatsu Flash4.0 V3 sCMOS camera. Widefield fluorescence excitation was performed with a Colibri 7-type RGB-UV fluorescence light source. The filter set was Zeiss set 90 LED.

### Image processing.

Images were processed with Zeiss Zen software applying default Airyscan processing followed by z-stack alignment if needed. Single slices, orthogonal projections, and three-dimensional (3D) renderings were produced in Zen software and exported as TIF files for assembly using Adobe Photoshop and Illustrator. Three-dimensional renderings were generated with default maximum settings in Zeiss Zen software.

### Infection by microinjection.

Yeast cells were grown from frozen stock on YPD plates at 30°C. The resulting stock plate was stored at 4°C and used for no more than 30 days. For microinjection, single colonies from 4°C stocks were inoculated into YPD medium and incubated with shaking overnight at 30°C. Overnight culture concentrations were diluted in phosphate-buffered saline (PBS) to an optical density at 600 nm (OD_600_) of 5.0 in a 1:10 dilution of phenol red. After manual dechorionation of embryos at ~28 hpf, intravenous (i.v.) inoculations were performed as previously described (49), with the alteration that larvae were positioned on a 3% agarose plate formed with holding grooves, as described in reference 41. Initial inoculum was documented by direct microscopic observation, and only larvae with initial inoculum between ~30 and 70 fluorescent yeast cells were used.

### Morpholino gene knockdown.

Paired PU.1 morpholinos were used as previously described (22). Morpholinos were synthesized and provided by Gene Tools, LLC (Philomath, OR, USA). PU.1 targeting sequences were CCTCCATTCTGTACGGATGCAGCAT (targeting the translation initiation site) and GGTCTTTCTCCTTACCATGCTCTCC (targeting the exon 4 to 5 boundary). Combined final concentrations were 0.375 mM and 0.025 mM, respectively. Control morpholino was the standard Gene Tools control with sequence CCTCTTACCTCAGTTACAATTTATA. Five nanoliters of the mixture was injected per embryo into the yolk at the 1- to 2-cell stage.

### Dextran assay of BBB integrity.

Zebrafish larvae at 4 dpi were microinjected via the tail vein with 10-kDa dextran conjugated to Alexa Fluor 647 (Invitrogen, D22914) at 25 mg/mL in PBS. Microscopic observations of yeast in the brain were made within 4 h of administration.
