# Supplementary material for: Roles for Microglia in Cryptococcal Brain Dissemination in the Zebrafish Larva
Source: Microbiol Spectr. 2023 Jan 31;11(2):e04315-22. doi: 10.1128/spectrum.04315-22 (PMC10100726; doi:10.1128/spectrum.04315-22)
Supplement: Supplemental file 1 — Fig. S1 to S6. Download spectrum.04315-22-s0001.pdf, PDF file, 0.6 MB [file spectrum.04315-22-s0001.pdf]

SUPPLEMENTARY FIGURES

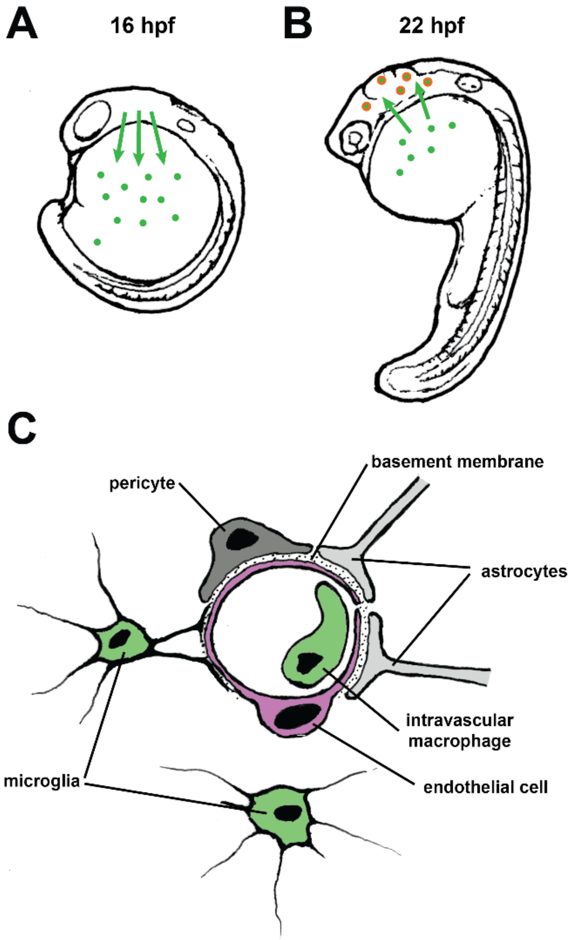

Fig. S1. Microglia, macrophages and the neurovascular unit of the zebrafish larva. A. The earliest developing macrophages in the larva arise from the dorsolateral mesenchyme and migrate under the periderm onto the superficial yolk ball around 16hpf. **B.** A subset of these early macrophages migrates into the developing brain around 22hpf, assuming a distinct gene expression pattern and becoming microglia around 72hpf. A. and B. are redrawn from reference 22. **C.** Schematic view of the neurovascular unit by 5dpf. Microglia are present in the brain parenchyma and often in contact with endothelial cells. Both microglia and macrophages are rendered in green to reflect their common expression of EGFP driven by the mpeg promoter. Note that stationary intravascular macrophages are seldom seen in the brain in the absence of infection. Pericytes and astrocytes are additional cell types, rendered in grey as they are not visualized in the experiments presented.

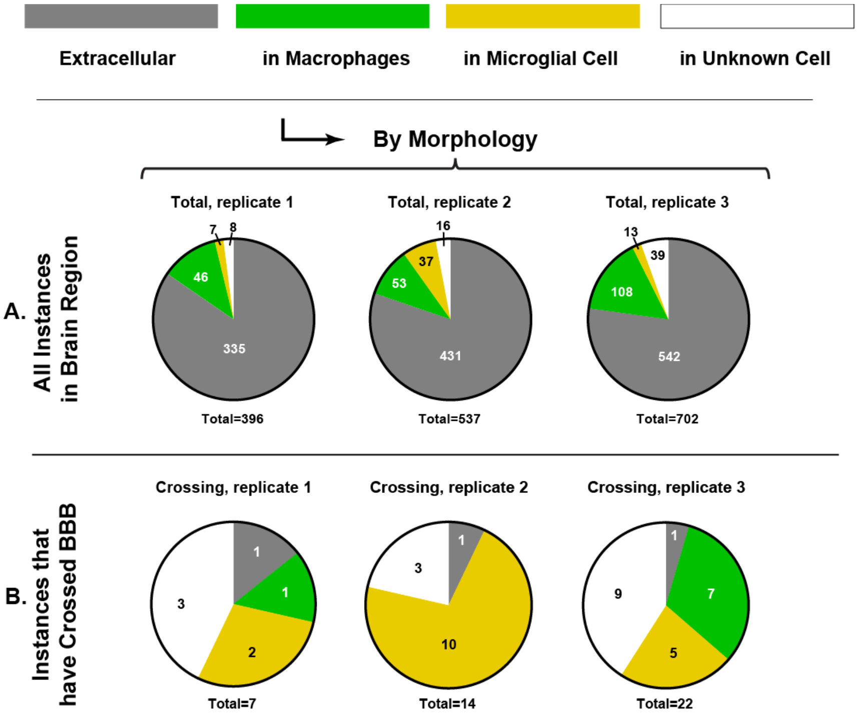

Fig. S2. Quantification of cryptococcal yeast cells in the brain, individual replicates. A. Characterization of instances of yeast in the brain of infected zebrafish larvae, with colors representing morphology. There were a total of 77 fish observed, with 27 fish in replicate 1, 26 fish in 2, and 24 fish in 3. **B.** Characterization of instances of yeast in the brain parenchyma, with the same colors and fish from A.

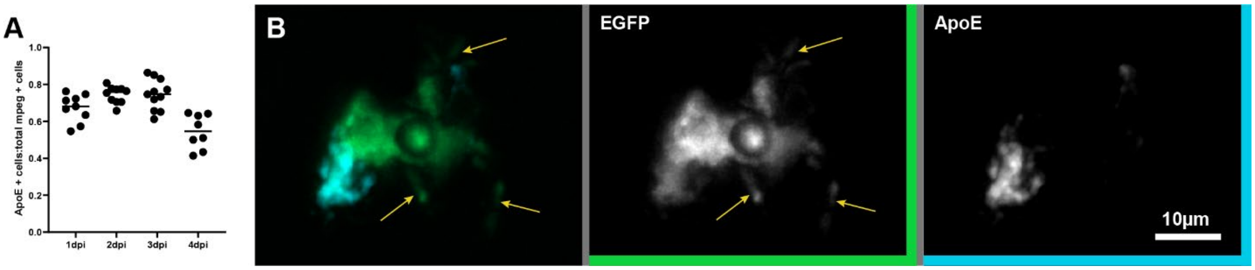

Fig. S3. Identification of microglial cells via in situ hybridization. A. Percentage of mpeg:EGFP+ cells in the brain of infected zebrafish larvae which also express *apoE*, by day. **B.** In situ hybridization for *apoE* (cyan) in infected *mpeg:EGFP* (green) larva. Yellow arrows indicate microglial ramifications.

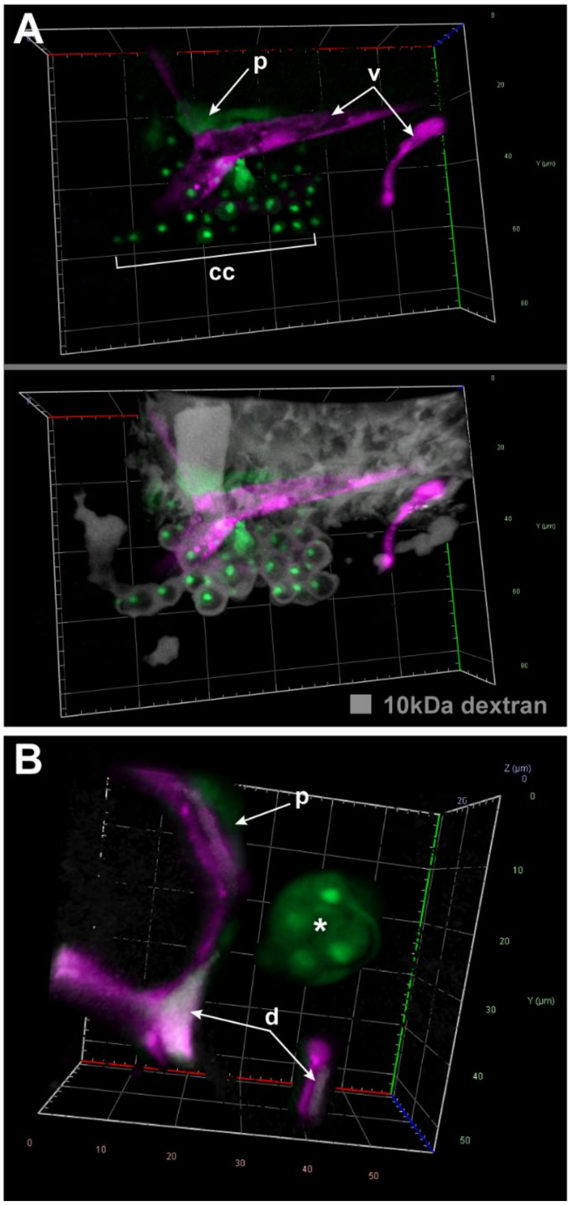

Fig. S4. CNS Vascular integrity during infection. A. Above: In a heavily infected larva, Cryptococcal cells (c) replicate in the parenchyma near intact vessels (v). A phagocyte (p) is located on the outside of the vessel. Below: 10kDa dextran (white/grey) leaks freely from the vessels and around the cryptococci. Grid 20µm. **B.** Dissemination into the parenchyma does not require vascular leakage. An infected phagocyte (*) is seen in the parenchyma next to vessels which contain dextran (d). Again a perivascular phagocyte (p), most likely a microglial cell, is seen in the vicinity. Green represents EGFP from mpeg-expressing host cells and *Cryptococcus*. Magenta is mCherry expressing endothelial cells. Grid 10µm.

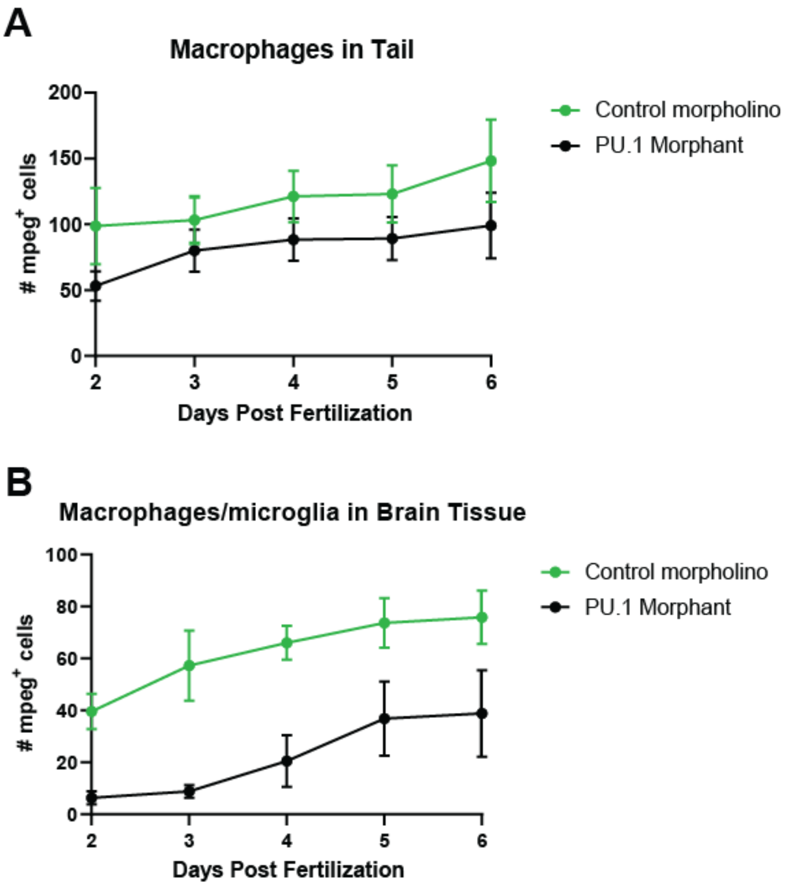

Fig. S5. Effect of PU.1 morpholino on phagocyte counts in head and tail. Graphs show daily absolute number of EGFP+ cells in *Tg(mpeg:EGFP)* larvae injected with PU.1 or control morpholino, in tail (A) and brain (B).

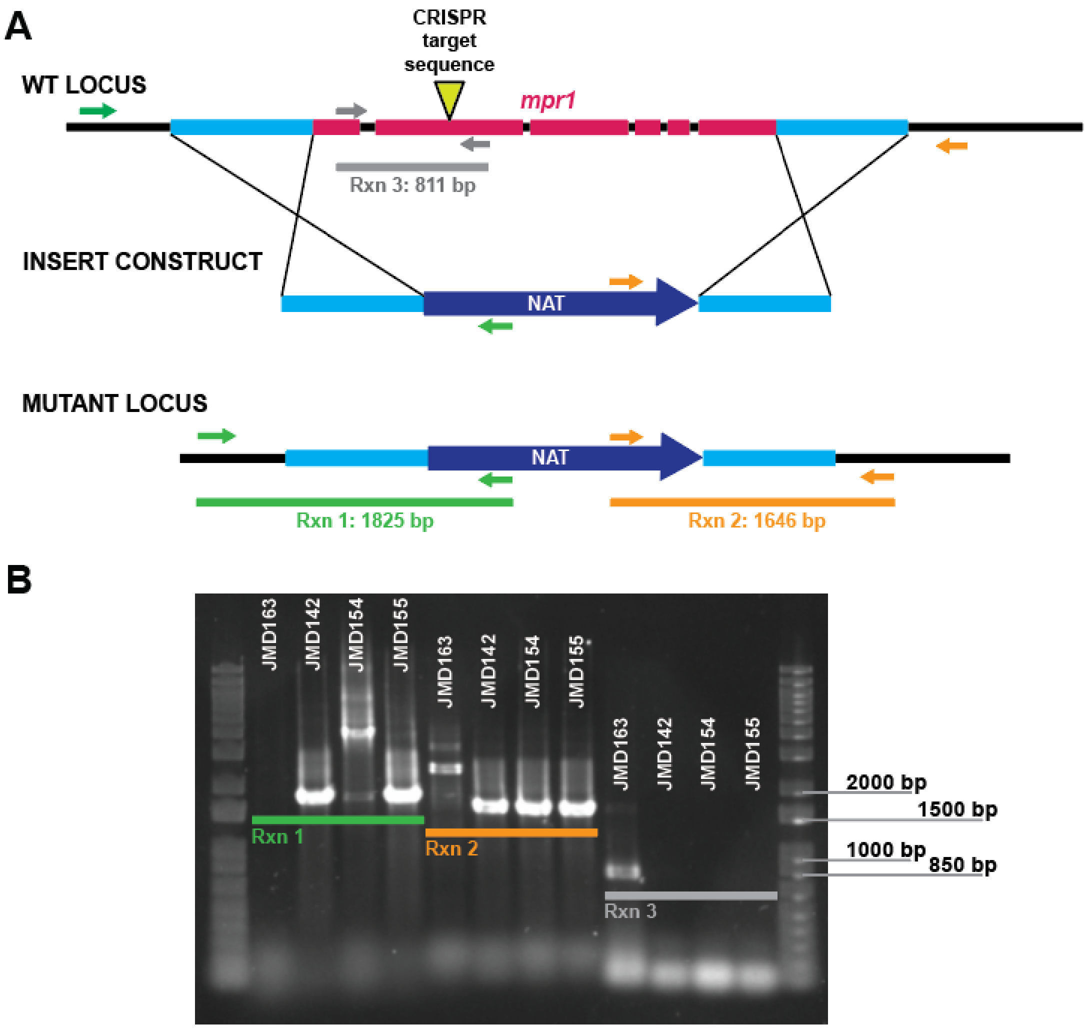

Fig. S6. Generation and testing of *mpr1Δ* mutant strains. **A.** Top: wildtype locus with *mpr1* exons in red and flanking regions in blue. 20mer CRISPR target locus noted with yellow triangle. Grey arrows and bar indicate screening PCR reaction #3. Middle: insert construct with nourseothricin resistance cassette (dark blue) between homologous flanking regions. Bottom: resulting targeted insertion of NAT cassette. Green and orange arrows and bars indicate screening PCR reactions #1 and 2. **B.** DNA electrophoresis of PCR reactions 1-3 for JMD163 (control), JMD142, JMD154 and JMD155 mutant strains. Bar colors represent the same reactions as in A.
